# ASSESSING “AGE-FRIENDLINESS” ON CAMPUS: LESSONS FROM GEORGIA STATE UNIVERSITY 50+ STUDENTS

**DOI:** 10.1093/geroni/igad104.2610

**Published:** 2023-12-21

**Authors:** Grace da Rosa, Mariam Qreshi, Jacqueline Laures-Gore, Hyunji Kim, Jennifer Morgan

**Affiliations:** Georgia State University, Atlanta, Georgia, United States; Georgia State University, Atlanta, Georgia, United States; Georgia State University, Atlanta, Georgia, United States; Georgia State University, Atlanta, Georgia, United States; Georgia State University, Atlanta, Georgia, United States

## Abstract

Older students (aged 50+) are an important, yet underrecognized group on today’s college campuses. Many campuses lack age-friendliness, which deters older students from enrolling and creates barriers for these students. In an effort to better understand age-friendliness of Georgia State University (GSU) campuses, we conducted a survey in Fall 2019 (n=411; 39% response rate). The survey assessed motivations for attending school, challenges on campus, perceptions of how the university is currently addressing their needs, factors/resources that helped adjustment to school, and the extent to which they experienced age discrimination on campus. Of the respondents, 45% were undergraduates (50% identified as Black; 38% identified as White); 41% were graduate students (29% identified as Black; 62% identified as White), and 14% were non-degree students (24% identified as Black; 62% identified as White). Thematic analysis of the responses suggest that the main challenges experienced by GSU older students are course-related (e.g., course load, bureaucracy getting enrolled, limited class options, scheduled course times, course modalities) and time-related (e.g., balance between school, work, and family). For example, students noted, “difficulties finding study groups/lab partners due to age differences” and “administrators not taking older students serious.” Technology barriers were pervasive in the open responses. Respondents further identified faculty/instructors as key resources for overcoming barriers. The survey served as an initial needs assessment to answer institutional questions informing the journey to becoming designated as an Age Friendly University. Implications for increasing age inclusivity and supporting older students will be discussed.

